# miR-486-5p expression is regulated by DNA methylation in osteosarcoma

**DOI:** 10.1186/s12864-022-08346-6

**Published:** 2022-02-17

**Authors:** Heidi M. Namløs, Magne Skårn, Deeqa Ahmed, Iwona Grad, Kim Andresen, Stine H. Kresse, Else Munthe, Massimo Serra, Katia Scotlandi, Antonio Llombart-Bosch, Ola Myklebost, Guro E. Lind, Leonardo A. Meza-Zepeda

**Affiliations:** 1 Department of Tumor Biology, Institute for Cancer Research, The Norwegian Radium Hospital, Oslo University Hospital, Oslo, Norway; 2 Department of Molecular Oncology, Institute for Cancer Research, The Norwegian Radium Hospital, Oslo University Hospital, Oslo, Norway; 3 Laboratory of Experimental Oncology, IRCCS Istituto Ortopedico Rizzoli, Bologna, Italy; 4 Department of Pathology, Valencia University, Valencia, Spain; 5 Department for Clinical Science, University of Bergen, Bergen, Norway; 6 Genomics Core Facility, Department of Core Facilities, Institute for Cancer Research, The Norwegian Radium Hospital, Oslo University Hospital, Oslo, Norway

**Keywords:** miRNA, Bone, cancer, Cell line, Epigenetic, Sarcoma, *ANK1*

## Abstract

**Background:**

Osteosarcoma is the most common primary malignant tumour of bone occurring in children and young adolescents and is characterised by complex genetic and epigenetic changes. The miRNA miR-486-5p has been shown to be downregulated in osteosarcoma and in cancer in general.

**Results:**

To investigate if the *mir-486* locus is epigenetically regulated, we integrated DNA methylation and miR-486-5p expression data using cohorts of osteosarcoma cell lines and patient samples. A CpG island in the promoter of the *ANK1* host gene of *mir-486* was shown to be highly methylated in osteosarcoma cell lines as determined by methylation-specific PCR and direct bisulfite sequencing. High methylation levels were seen for osteosarcoma patient samples, xenografts and cell lines based on quantitative methylation-specific PCR. 5-Aza-2′-deoxycytidine treatment of osteosarcoma cell lines caused induction of miR-486-5p and *ANK1*, indicating common epigenetic regulation in osteosarcoma cell lines. When overexpressed, miR-486-5p affected cell morphology.

**Conclusions:**

miR-486-5p represents a highly cancer relevant, epigenetically regulated miRNA in osteosarcoma, and this knowledge contributes to the understanding of osteosarcoma biology.

**Supplementary Information:**

The online version contains supplementary material available at 10.1186/s12864-022-08346-6.

## Introduction

High-grade osteosarcoma is the most prevalent primary malignant tumour of bone, affecting both children, adolescents and, more rarely, elderly people. Following the introduction of multi-agent chemotherapy in the 1970s, the 5-year survival rate increased considerable reaching 60–70% among patients with conventional high-grade osteosarcoma [1]. However, advances in treatment have stalled with no further improvement in the survival [2], and also exhibit a collateral risk for adverse toxicity events. Improved biological knowledge is required to develop new treatment opportunities and further improve the survival of osteosarcoma patients.

Osteosarcoma is characterized by considerable phenotypic and genomic heterogeneity, and few recurrent targetable genetic changes have been reported. Osteosarcoma exhibits a complex karyotype with high genetic and chromosomal instability seen as multiple rearrangements across the genome, kataegis and chromothripsis [3–6]. The genetic markers identified have been associated with treatment response and prognosis, thus appearing as promising candidates for a translation to clinical practice [7, 8]. However, the limited extent of recurrent profiles identified indicates that a substantial regulation of the transcriptional programs in osteosarcoma may rather be caused by epigenetic programs [9–13], providing novel avenues for cancer therapy [14].

Epigenetic mechanisms are fundamental drivers of tumour initiation, development and progression [15]. Several epigenetic alterations have been identified, including biomarkers for various diseases [16]. DNA methylation is the most commonly studied epigenetic alteration in cancer [17], comprising covalent addition of methyl groups to CpG sites. CpG islands (CGIs) are characterized by regional enrichment of CpG sites and are present in approximately 70% of all human gene promoters [18]. In normal cells, these CGIs are usually unmethylated. In cancer, however, CGI hypermethylation is frequently observed, accompanied by long-term silencing of gene expression [19].

MicroRNAs (miRNAs) are a key class of epigenetic regulators as they act to post-transcriptionally silence large numbers of genes without modifying the DNA [20]. miRNAs are frequently associated with CGIs and are themselves also found silenced through epigenetic mechanisms [21]. Dysregulated miRNA expression may result in aberrant expression of genes that play critical roles in osteosarcoma tumorigenesis and progression [22–25]. We have previously identified miR-486-5p to be among the most downregulated miRNAs in osteosarcoma cell lines [24]. miR-486 has been shown to be downregulated in osteosarcoma patient samples compared to normal samples [26]. However, the mechanism of repression of miR-486-5p is still unknown in osteosarcoma. Given that epigenetic regulation of miR-486-5p has been described in other cancers like lung cancer [27], we hypothesized that this could be the mechanism of miR-486-5p regulation also in osteosarcoma.

In an effort to advance the understanding of osteosarcoma biology, we aimed to investigate if the expression of miR-486-5p was epigenetically regulated through methylation of promoter regions. We analysed miR-486-5p expression and DNA methylation levels in a cohort of osteosarcoma cancer cell lines and patient samples. Qualitative and quantitative methylation analyses were performed, allowing us to characterize the *mir-486* locus in detail. Finally, the in vitro effect of miR-486-5p overexpression on cell morphology and proliferation was investigated.

## Results

### Low expression of miR-486-5p in osteosarcoma

**Fig. 1:**
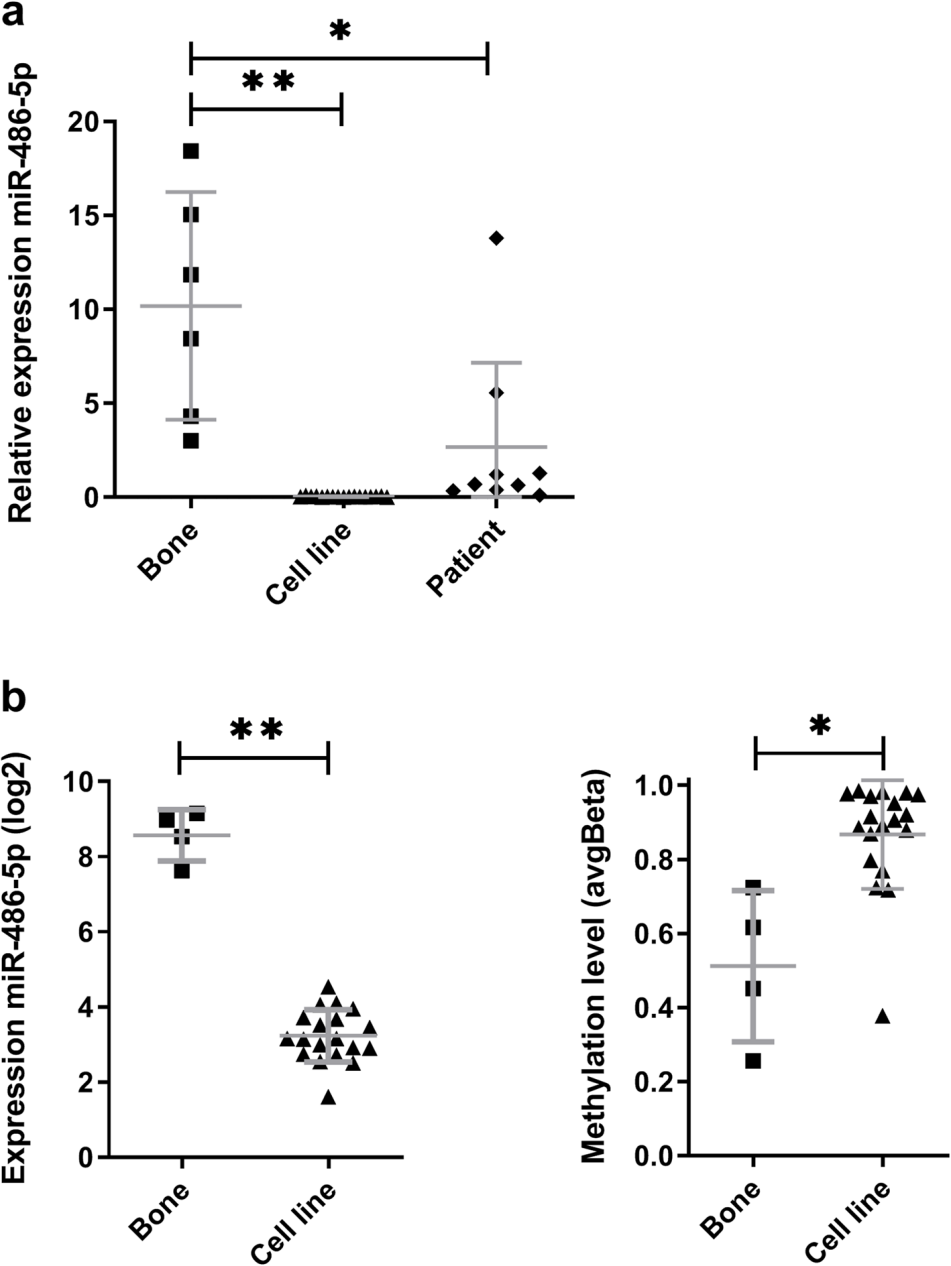
miR-486-5p expression and methylation in osteosarcoma cell lines and patient samples. **A**. miR-486-5p expression level in normal bone (*n* = 6), cell lines (*n* = 17) and patient samples (*n* = 9) using qRT-PCR. The expression level was quantified for the groups of samples. The values are shown relative to mean expression of bone. **B**. DNA methylation and miRNA expression levels in normal bone samples (*n* = 4) and osteosarcoma cell lines (*n* = 19) using arrays. The expression level of miRNAs (log2) and DNA methylation level (Beta, probe cg00176210) were obtained using Agilent miRNA array v2 and Illumina Infinium Methylation27 BeadChip technology, respectively. Values are given as mean (SD) with whiskers from min to max. *P*-values * < 0.01 and ** < 0.0001

To follow up on our earlier observations of miR-486-5p in osteosarcoma [24], miR-486-5p expression level was examined in a panel of osteosarcoma patient samples (*n* = 9), osteosarcoma cell lines (*n* = 17) and normal bone samples (*n* = 6) by quantitative real-time reverse-transcription PCR (qRT-PCR). The mean expression of miR-486-5p was reduced 3-fold in patient samples (*p* < 0.01) and 300-fold in cell lines (*p* < 0.0001) compared to normal bone (Fig. 1a). This confirms a low expression of miR-486-5p in osteosarcoma using both patient tissue samples and cell lines.

### DNA methylation is associated with low expression of miR-486-5p in osteosarcoma cell lines

*miRr-486-5p* is encoded within the last intron of the Ankyrin 1 (*ANK1*) gene*.* Several transcript variants of human *ANK1* exist, with multiple transcriptional start sites (TSS), promoter regions and CGIs. The *ANK1* variants 1–4 represents medium-sized transcripts, *ANK1* variant 9 is a longer transcript while *sANK1* represents the short variants 5, 7 and 10 (Additional file 1: Supplementary Fig. S1). The methylation level of *ANK1* was quantified in osteosarcoma cell lines (*n* = 19) and normal bone samples (*n* = 4) using Illumina HumanMethylation27 BeadChips. This microarray contains one CpG site in the CGI CpG79 (chr8:41654876–41,655,984) at the TSS (position chr8:41655140) of *ANK1* (variant 1–4). The average difference of methylation in cell lines versus normal bone samples was 0.36 (DeltaBeta) for CpG79, providing a first evidence for methylation of the *mir-486* locus.

We next compared the expression and methylation level of miR-486-5p for the above sample cohort. The expression level of miR-486-5p was quantified using Agilent miRNA array v2. The mean expression of miR-486-5p was significantly lower in cell lines than in bone (*p* < 0.0001) and the methylation was higher in cell lines than in bone (*p* < 0.01). Taken together, this indicated an association between low expression of miR-486-5p and CpG methylation of *ANK1* (Fig. 1b).

### Demethylation caused increased expression of miR-486-5p

**Fig. 2:**
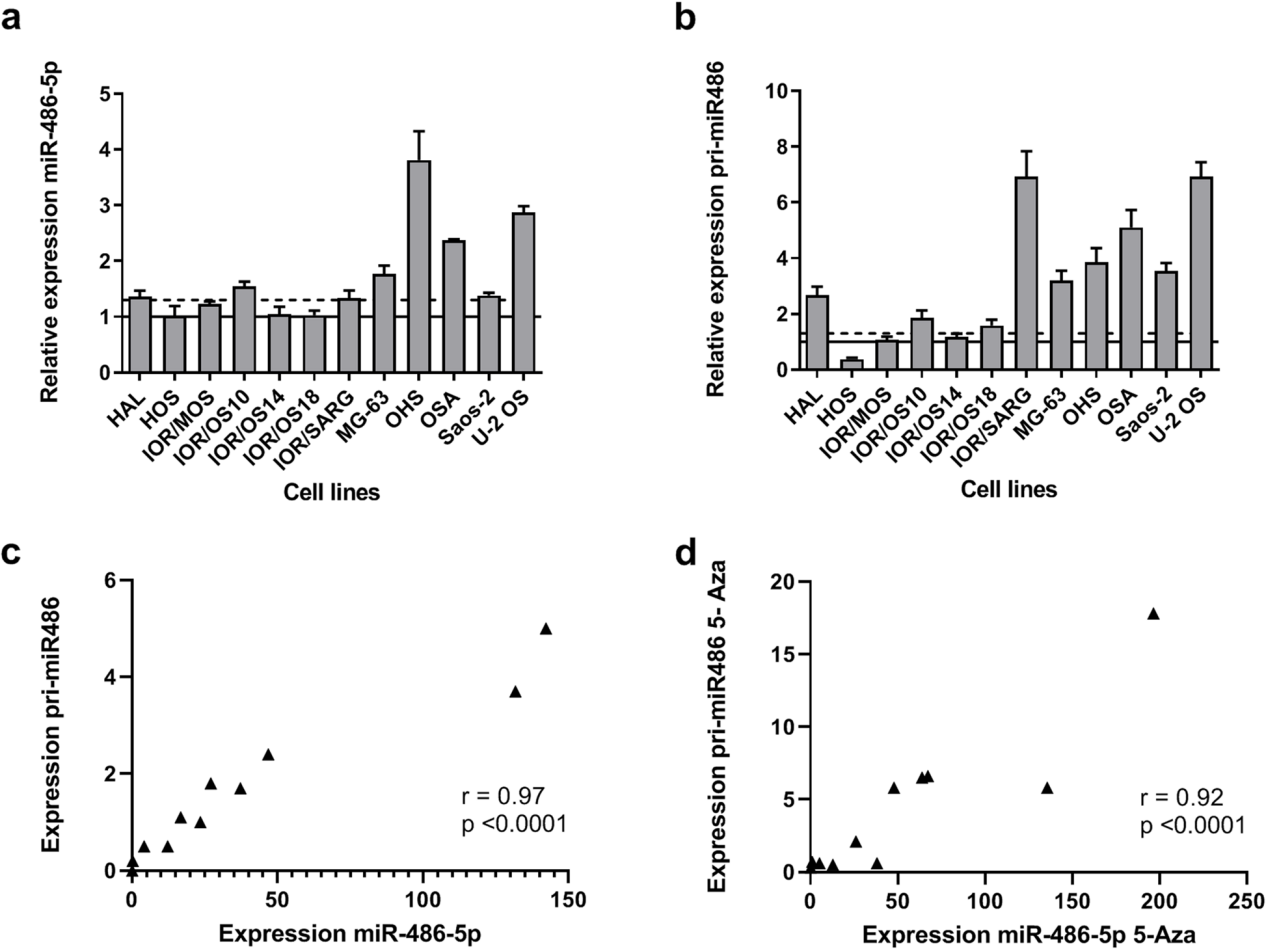
Expression and correlation of miR-486-5p and hsa-mir-486-1 (pri-mir486) in osteosarcoma cell lines upon 5-Aza-2′-deoxycytidine treatment. Relative expression level **A.** miR-485-5p and **B.** pri-mir486 after 72 h of 5-Aza treatment**.** The values are shown relative to treated cell lines (set to 1, horizontal line). Induction of > 30% shown as dotted line. Values are given as mean (SD). Correlation between expresion level of miR-485-5p and pri-mir486 in **C.** untreated and **D.** 5-Aza treated cell lines. The expression levels are quantified using qRT-PCR, and normalized against *RNU44* for miR-486-5p and *GAPDH* for pri-mir486

We next examined the effect of changes in methylation on miR-486-5p expression. Osteosarcoma cell lines (*n* = 12) were treated with the demethylation agent 5-Aza-2′-deoxycytidine (5-Aza). After 72 h, the relative expression levels of miR-486-5p in untreated and treated cell lines were quantified by qRT-PCR. A significant difference between the cells before and after treatment was observed (*p* = 0.02). miR-486-5p showed at least a 1.3-fold (30%) induction in 8/12 tested cell lines (Fig. 2a), indicating that the expression of miR-486-5p was affected by changes in DNA methylation.

To exclude that the expression level of mature miR-486-5p was affected by post-transcriptional processing, the pri-miRNA hsa-mir-486-1 (pri-mir486) was quantified by qRT-PCR. pri-mirR486 showed at least 1.3 fold (30%) induction in 9/12 cell lines after 5-Aza treatment, confirming the epigenetic effects seen on the mature miRNA (Fig. 2b). A high correlation with was observed between mature miR-486-5p and the pri-miRNA, with Pearson’s correlation r = 0.97 (*p* < 0.0001) before 5-Aza treatment and r = 0.92 (*p* < 0.0001) after treatment of the cell lines (Fig. 2c and d).

### The genes within the *mir-486/ANK1* locus were observed to be co-regulated

To address the relationship between expression of miR-486-5p and the different *ANK1* transcript variants, transcript-specific qRT-PCR was performed. The human *ANK1* variant 9 has the most upstream first exon, whereas an alternative exon 1 is used in variants 1–4. *sANK1* represents the short variants 5, 7 and 10, with an alternative exon 1, while exons 41 and 42 are in common with the other transcript variants (Additional file 1: Supplementary Fig. S1).

**Fig. 3:**
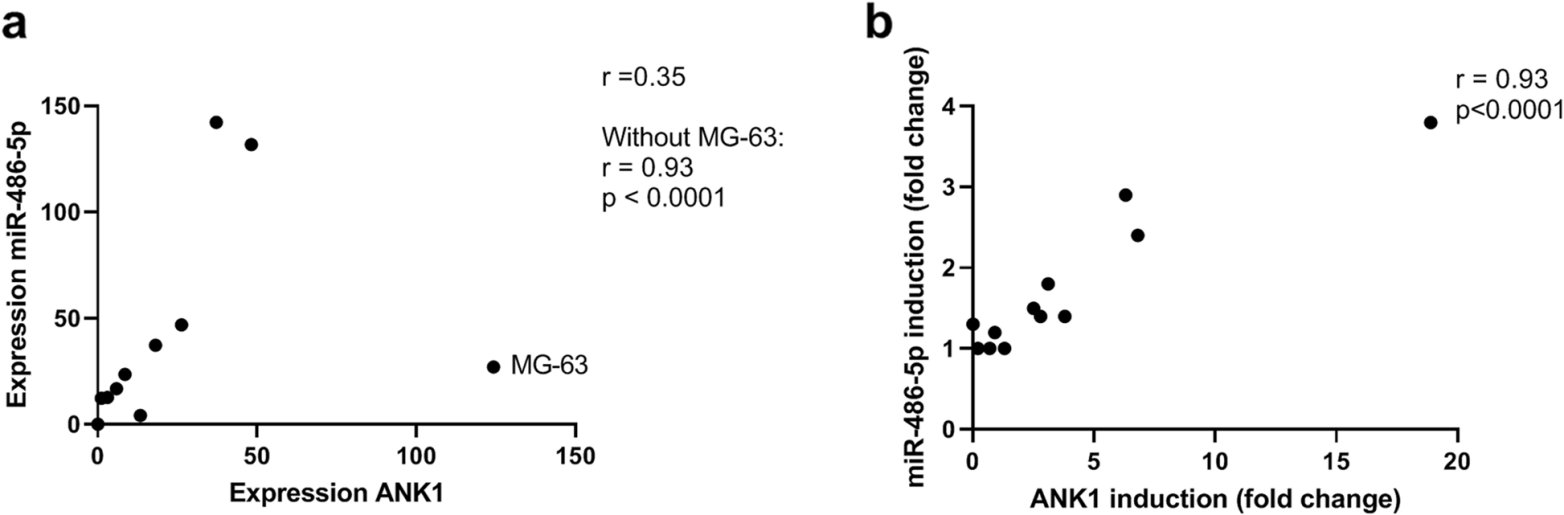
Expression and correlation of miR-486-5p and *ANK1* before and after 5-Aza-2′-deoxycytidine treatment in osteosarcoma cell lines. **A**. Quantification of expression of miR-486-5p and *ANK1* versions 1–4 in untreated cells. **B**. The induction of miR-486-5p and *ANK1* versions 1–4 after 5-Aza treatment. The induction fold change is calculated as the ratio between expression of untreated and 5-Aza treated transcripts as quantified by qRT-PCR. The expression is normalized against *RNU44* for miR-486-5p and *GAPDH* for *ANK1*. Pearson’s Correlation r is calculated with and without the outlier sample MG-63

In osteosarcoma cell lines, *ANK1* variants 1–4 were found to be moderately expressed in 8/12 lines. The *sANK1* variant was only weakly detected in 3/12 cell lines, while variant 9 was undetected (Additional file 1: Supplementary Fig. S2). Comparing expression levels of miR-486-5p and *ANK1*, no similarity was observed between miR-486-5p and sANK1 or *ANK1* variant 9. For miR-486-5p and *ANK1* variants 1–4, a high correlation (Pearson’s correlation r = 0.93, *p* < 0.0001) could be observed across the cell line panel if one of the cell lines, MG-63, was omitted (Fig. 3a). Similar observations were done for pri-miR486 (Pearson’s correlation r = 0.92, *p* < 0.0002). The cell line MG-63 showed very high expression levels of *ANK1* variants 1–4, however as the sample seems to be a biological and not a technical outlier, it was not removed from the dataset.

*ANK1* variant 1–4 expression was induced in 8/12 cell lines upon 5-Aza treatment (Additional file 1: Supplementary Fig. S2). Comparing the effect of 5-Aza treatment on the mRNA level and miRNA level, it was observed that all 7 cell lines with increased expression of miR-486-5p also had increased expression of *ANK1* variant 1–4. The level of induction was similar for *ANK1* and miR-486-5p, and a high correlation (Pearson’s Correlation r = 0.93, *p* < 0.0001) could be seen between the fold change observed for *ANK1* and the fold change observed for miR-486-5p upon Aza treatment across all 12 cell lines (Fig. 3b). No correlation between miR-486-5p and *ANK1* induction was observed for the other *ANK1* variants. The *sANK1* variant was only induced in four cell lines and no variant 9 transcripts could be detected upon 5-Aza treatment (Additional file 1: Supplementary Fig. S2). These observations support a co-regulation between miRNA and mRNA genes in the *miR-486/ANK1* locus.

### Genome-wide methylation profiling revealed hypermethylation of a CGI upstream of *mir-486* in osteosarcoma patient samples

**Fig. 4:**
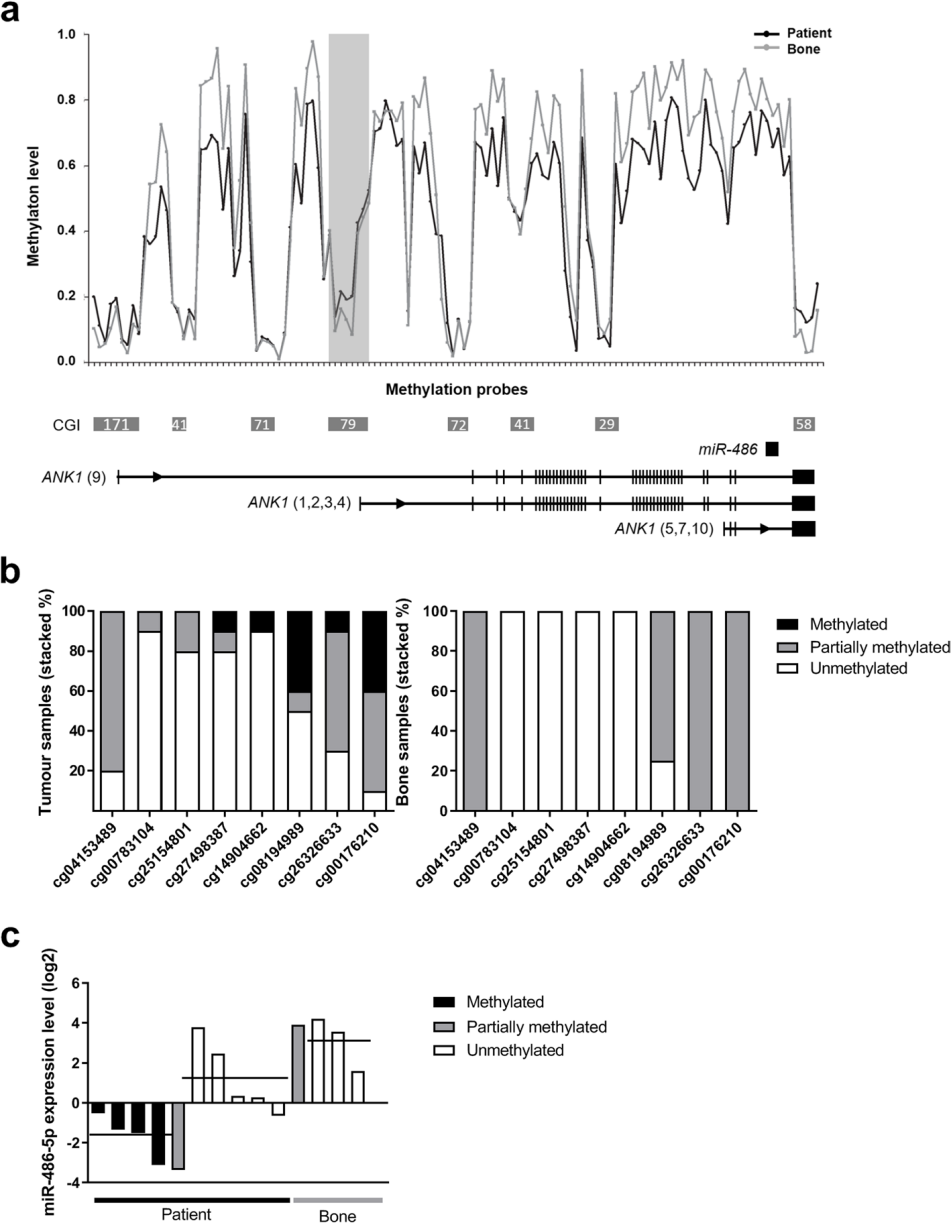
Genome-wide methylation profile of the *mir-486/ANK1* locus and miR-486-5p expression levels in osteosarcoma patient samples. **A.** Representation of average methylation profile across the *mir-486*/*ANK1* locus in osteosarcoma patient samples (*n* = 10) and bone (*n* = 4). Methylation level (Beta) is shown for the individual CpG sites (ticks on horizontal axis) from the HumanMethylation450 BeadChips. The probes are ordered along the locus and intervals are not in scale. CGIs are shown as grey boxes with number refering to CpG count (from UCSC Genome Browser NCBI, GRCh37/hg19 assembly). Horizontal lines below plot show representative transcript variants of the respective mRNA genes (RefSeq) with exons as vertical bars, not in scale. Shaded vertical box: CGI shown in detail in B. Black, osteosarcomas; Grey, bone. **B.** Probe level methylation for CGI CpG79 in osteosarcoma patient samples and bone. Methylation levels as determined by Infinium 450 k arrays are given in Beta values for the individual CpG sites (cg). The selected CpGs within CGI CpG79 are highlighted with a shaded vertical box in A. Methylated: Beta 0.7–1.0; partially methylated: Beta 0.3–0.7; unmethylated: Beta< 0.3. **C.** Expression level of miR-486-5p in osteosarcoma patient samples and bone based on qRT-PCR. The osteosarcoma samples are grouped based on methylation status for probe cg08194989 on Infinium 450 k arrays, while the bone samples are shown as one group. Horizontal line: mean value

To investigate the methylation level of the whole *mir-486* locus, we performed high-resolution Infinium 450 k methylation array analysis on a set of 10 osteosarcoma patient samples and four normal bones. For the *mir-486/ANK1* locus, methylation data from 96 CpG sites across all CGIs were determined. The CpG sites located in non-CGI regions were in general hypermethylated in both groups of samples, with a higher level for bone. Higher methylation levels for osteosarcoma patient samples compared to bone were only observed in the CGI CpG79 (Fig. 4a), showing different levels of methylation within the patient cohort. The bone samples show a similar pattern of methylation across the CGIs, but with lower level of methylation with only partially methylated and unmethylated sites (Fig. 4b).

The patients samples were classified as unmethylated or methylated based on methylation level of one of the CpG sites that had the most evident methylation in CGI CpG79. In general, miR-486-5p was observed to be low expressed in highly methylated osteosarcoma samples and higher expressed in unmethylated samples with *p* = 0.03. The bone samples showed the same level of expression as unmethylated osteosarcoma samples (Fig. 4c). This suggests that methylation of an upstream regulatory region affects miR-486-5p expression in osteosarcoma patients.

### Qualitative and quantitative methylation analysis confirms hypermethylation of *mir-486* in osteosarcoma

**Fig. 5:**
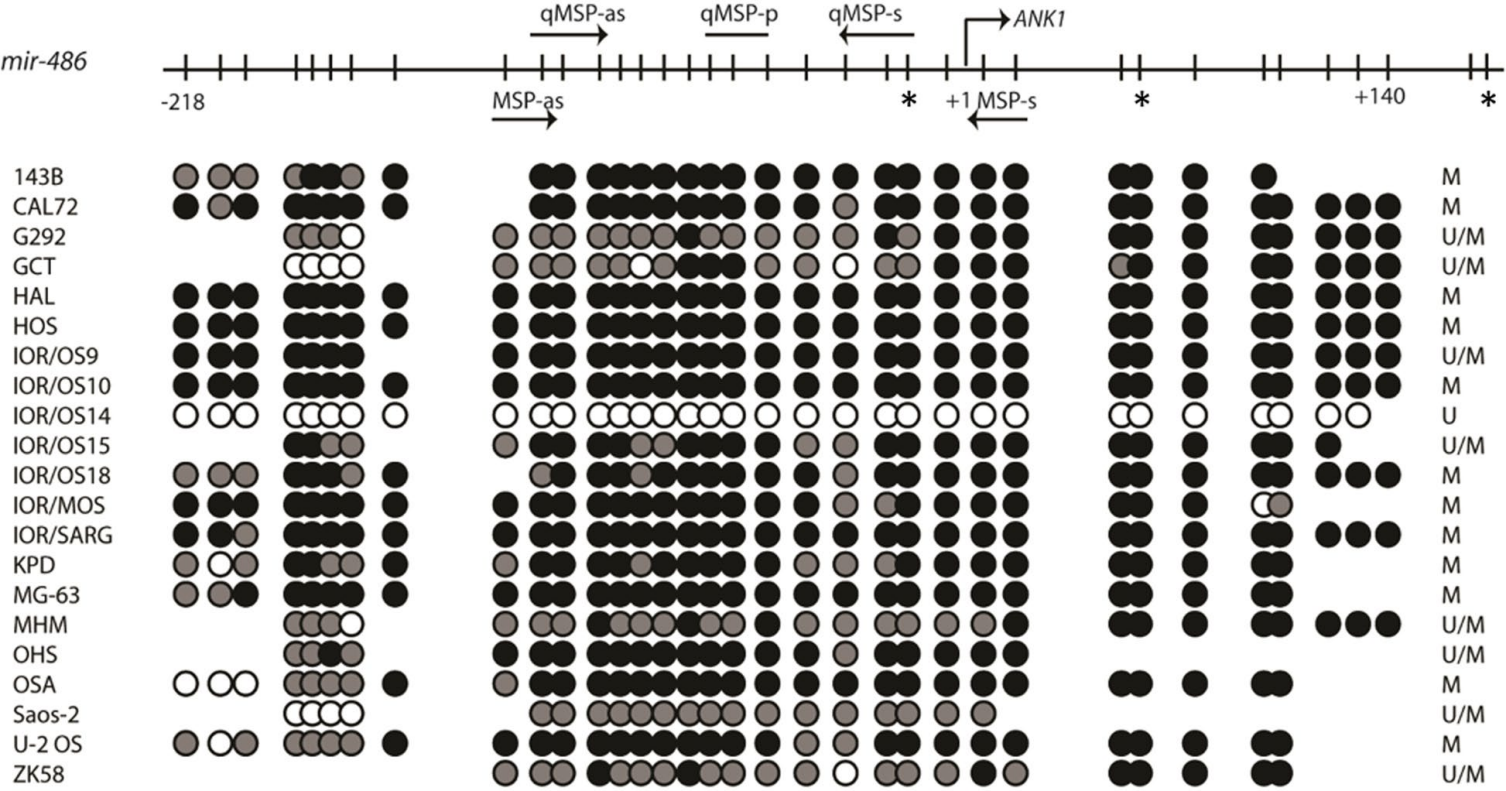
Cell line methylation status of CGI CpG79 located upstream of *mir-486* as assessed by methylation specific PCR and direct bisulfite sequencing. The upper part is a schematic presentation of the CpG sites (vertical bars) amplified by the bisulfite sequencing primers. The transcription start site (+ 1) refers to the start of *ANK1* (transcript variants 1–4). Arrows indicate location of MSP and qMSP primers, * represents the CpGs covered by the Infinium 450 k methylation array (cg08194989, cg26326633, cg00176210, respectively). For the lower part of the panel, black circles represent methylated CpGs (> 80% cytosine); grey circles represent partially methylated sites (20–80% cytosine) and white circles represent unmethylated sites (< 20% cytosine). The column to the right lists the methylation status of the respective cell lines as assessed by MSP analyses

Qualitative methylation-specific polymerase chain reaction (MSP) analysis was done for CGI CpG79 and CpG171, both located upstream of *mir-486*. The CGI CpG79 was hypermethylated in 20/21 cell lines (Fig. 5). Interestingly, this CGI was unmethylated in the IOR/OS14 cell line, previously shown to be globally hypomethylated [10]. The CGI CpG171 was only found to be hemimethylated in one cell line, and unmethylated in all the others (Additional file 1: Supplementary Fig. S3). Thus, further focus was done on the CGI CpG79.

Direct bisulfite sequencing was performed on osteosarcoma cell lines. The analysis was done on an extended region of the CGI CpG79, covering a total of 34 CpGs including the region for the MSP on CpG79 (Fig. 5). The resulting data confirmed the methylation status previously determined by MSP.

**Fig. 6:**
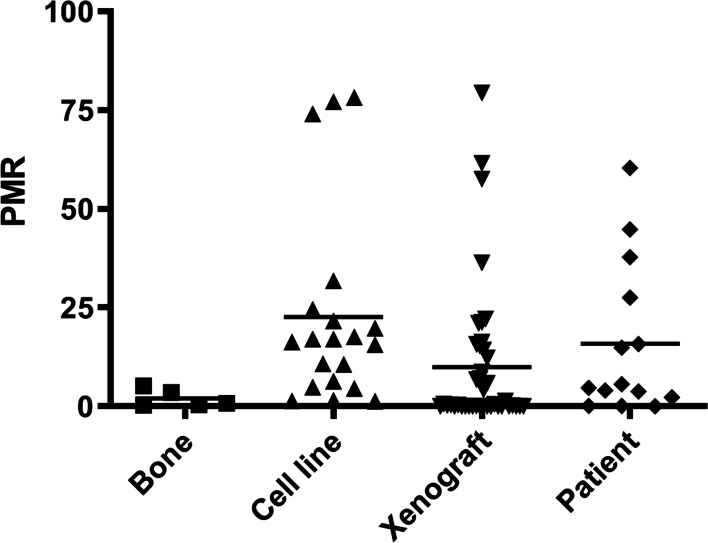
Methylation distributions in osteosarcoma assessed by quantitative methylation-specific PCR. The methylation was measured for the groups of normal bones and osteosarcoma cell lines, xenografts and patient samples. PMR, percent of methylated reference. Horizontal black line: mean values

The methylation of a region of CpG79 of *mir-486* was analysed quantitatively by quantitative methylation-specific polymerase chain reaction (qMSP) in a larger cohort of samples, including osteosarcoma cell lines (*n* = 20), xenografts (*n* = 41), patient samples (*n* = 14) and bone (*n* = 5). Methylated percent of methylated reference (PMR) was set to > 5.2. High qMSP methylation pattern was only observed in cancer samples, comprising both cell lines, xenografts and patient samples (Fig. 6). Mann–Whitney U test was used to compare the PMR values of the candidate regions in the different tissues, showing only significant differences between bone and cell lines (*p* = 0.005). For the cell lines, 17/20 (85%) showed increased methylation (>2x) compared to the bone average. However, increased methylation was also observed for 8/14 (60%) of the patient’s samples and 17/41 (40%) of the xenografts. Together, these observations suggest that the CGI CpG79 is highly methylated in osteosarcoma.

### Overexpression of miR-486-5p induces morphological changes in osteosarcoma cell lines

**Fig. 7:**
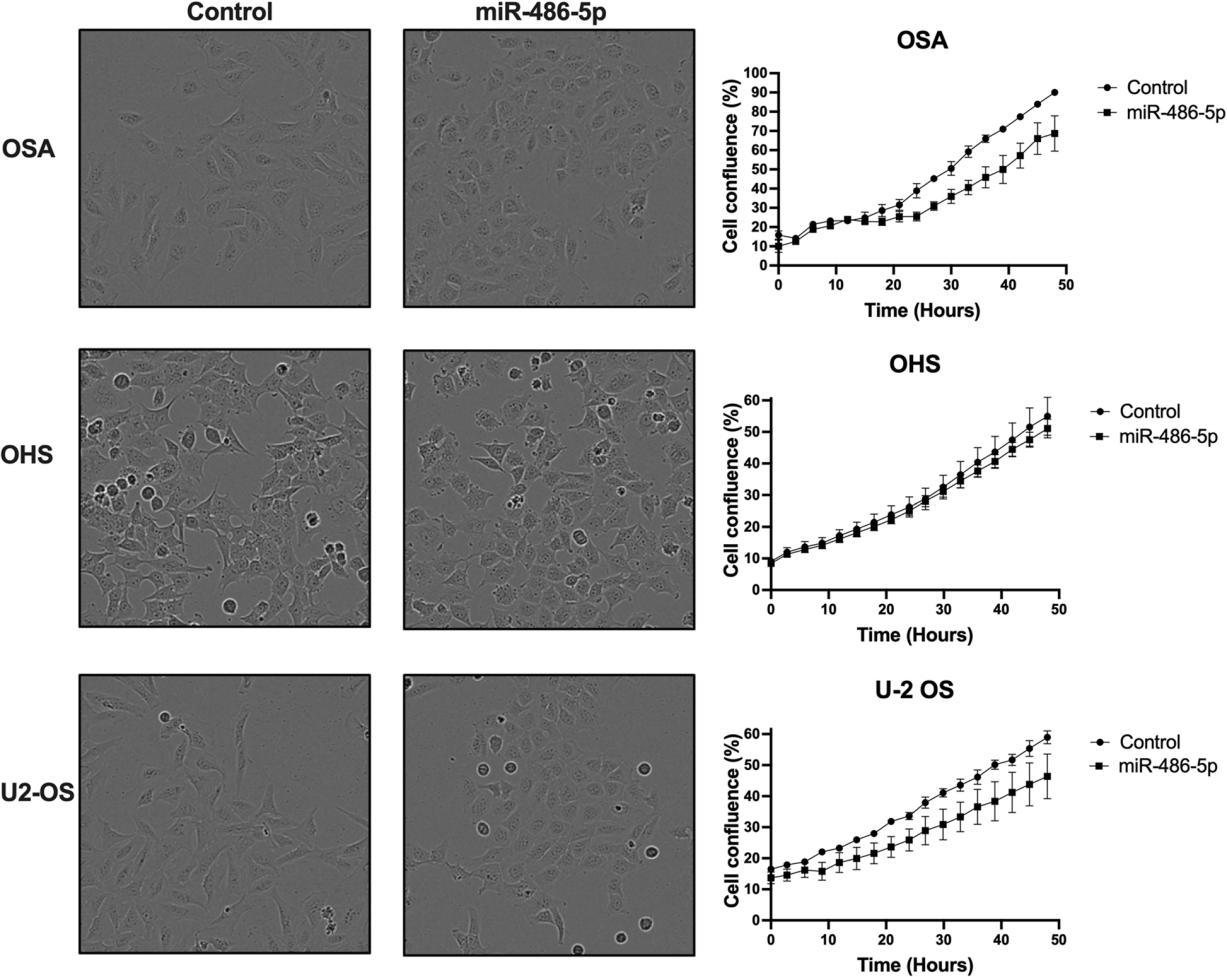
Introduction of miR-486-5p in osteosarcoma cell lines. Osteosarcoma cells (OSA, OHS and U-2 OS) were transiently transfected with synthetic miR-486-5p mimics or a negative control. Cellular proliferation rates were determined by live cell imaging for 48 h using the IncuCyte, measuring cell confluence over time. One representative experiment of three is shown (*n* = 3). Error bars represent the standard error of means of values for replicate wells (*n* ≥ =5)

To investigate the in vitro effect of miR-486-5p overexpression, we transiently transfected the osteosarcoma cell lines OSA, OHS and U-2 OS with miR-486-5p synthetic miRNA mimics. These cell lines showed the highest level of induction upon 5-Aza treatment. The iIntroduction of miR-486-5p caused a reduced growth rate in OSA and U-2 OS at 48 h, although the changes were not statistically significant (*p* = 0.2). However, a clear change in cell morphology was observed in all three cell lines where cells became flatter, rounder and smaller (Fig. 7). Thus, changes in miR-486-5p levels seem to affect the osteosarcoma cell phenotype.

## Discussion

Epigenetic mechanisms such as DNA methylation play crucial roles in controlling miRNA gene expression. Based on our previous observations of a strong, cancer-specific downregulation of miR-486-5p in osteosarcoma cell lines [24], recently confirmed in osteosarcoma patients [26], we set out to investigate the epigenetic regulation of miR-486-5p in osteosarcoma.

In this study, we have shown that the expression of miR-486-5p is differently regulated in osteosarcoma compared to normal bone. A heterogeneity was seen among the patient cohort regarding methylation status. The patients seem to be divided in two groups, one group highly methylated with low expression and a second group with an opposite pattern of low methylation and high expression. One might speculate that the miRNA regulation can be associated with disease aggressiveness, but extensive clinical data was not available for all of the patients in the cohort.

*mir-486* is located intragenic within the last intron of *ANK1*, which encodes ankyrin-1*.* The *mir-486*/*ANK1* locus is complex, and contains multiple promoter regions and CGIs. Alternative splicing and distinct use of promoters give rise to different isoforms of *ANK1* [28, 29]. The methylation of the CGI covering the TSS of *ANK1* variants 1–4 indicates that the *mir-486* locus is under control of the *ANK1* gene promoter in this region. This CGI was the only CGI in this locus that was hypermethylated in a high-resolution analysis, and cancer-specific methylation was verified in cancer cell lines, xenografts and clinical samples. In many cases, intragenic miRNAs are co-expressed with their host genes, suggesting that they depend on the same regulatory mechanisms [30, 31]. The similar expression patterns between the pri-miRNA, the mature miR-486-5p and *ANK1* variants 1–4 indicate that the miRNA and mRNA of the *mir-486/ANK1* locus is transcribed together. Upon demethylation treatment, the expression levels of pri/mature miR-486 and *ANK1* changed at similar levels across the osteosarcoma cell line panel, supporting a common epigenetic regulation from the *ANK1* variant 1–4 gene promoter.

It has been shown that alternative promoters give individualized regulation of transcripts with distinct first exons, providing an expression pattern with a strong tissue preference [32]. The regulation of the *mir-486/ANK1* locus may depend on the specific cellular context. The putative host gene *ANK1* is a prototype of the ankyrin membrane proteins, linking integral membrane proteins to the underlying spectrin network in erythroid cells [33], and has also been found in brain and muscles [28, 34]. The diversity of the ankyrins suggests that the isoforms might serve different roles in various cell types. The *ANK1* variant 1–4, observed to be co-expressed with miR-486-5p in our study, has previously been shown to be relevant for miR-486-5p expression in leukaemia. In myeloid leukaemia with an erythroid phenotype, miR-486-5p was regulated through *GATA1* binding to the promoter region of *ANK1* variant 1–4 [35]. An early study showed that mir-486-5p can be controlled by an alternative, muscle-specific promoter within intron 40 of the *sANK1* variant [36]. The *sANK1* isoform could only be detected for a few of the osteosarcoma cell lines in our study and was only induced in a subset of the cell lines. An extensive study of miR-486-5p was recently performed in non-small cell lung cancer, showing that miR-486-5p was co-expressed with *ANK1* variant 9 in lung cancer and lung epithelial cells. Aberrant methylation of the CGI covering the TSS of *ANK1* variant 9 (termed ANK1B promoter) repressed both *ANK1* and miR-486-5p [27], and was specific for adenocarcinoma. In our study, this CGI was unmethylated in all the osteosarcoma cell lines and the transcript levels were not induced upon 5-Aza treatment.

The induction of miR-486-5p expression in osteosarcoma cells resulted in a change in cell morphology, as well as a pattern of reduced cell proliferation in 2 out of 3 cell lines tested. A morphological change in MG-63 cells following overexpression of miR-486-5p has been described previously, possibly related to an EMT-like phenotype [37]. Through in vitro experiments, it has been shown that miR-486 can reduce proliferation, promote apoptosis and inhibit metastasis through regulation of the PKC-δ pathway in osteosarcoma cells [26]. miR-486 also promotes pro-osteogenic activity through induction of myofibroblastic differentiation through the PTEN–AKT pathway [38]. Following our and others observations of a low expression level of miR-485-5p in cancer, miR-486-5p has been described as a tumour suppressor in breast carcinoma, colorectal cancer, oesophagal cancer hepatocellular carcinoma, lung cancer, gastric carcinoma, myxoid liposarcoma, colorectal cancer, oesophagal cancer and lately leukemia [27, 39–46]. The functions of miR-486-5p in cancer cells are controversial, and other reports show that miR-486-5p rather play a causative, oncogenic role, as in other solid tumours like gliomas, prostate and cervical cancer, through negative regulation of multiple tumour suppressor pathways [47–49].

There were a couple limitations to our study. The sample size of the normal samples is smaller than the included osteosarcoma samples. Furthermore, the manuscripts only showed that the hypermethylation in the differentially methylated region correlated with downregulation of miR-486-5p expression. Although a direct effect is not proven, an inverse pattern between methylation and gene expression is shown and the other results performed support the observation that the miRNA is epigenetic regulated. Further investigations regarding the epigenetic regulation of *mir-486* in various tissue types would be highly interesting.

In conclusion, the present study of miR-486-5p shows that the low expression observed in osteosarcoma is caused by cancer-specific methylation of the upstream promoter region of *ANK1* variant 1–4. This implies a tumor suppressive role of miR-486-5p in osteosarcoma, and the findings may lead to clarify the tissue/disease-specific regulation of the miR-486-5p expression. The main effect of miR-486-5p overexpression was morphological changes in osteosarcoma cells. The epigenetic regulation should be further correlated with clinical characteristics, and the functional role needs to be further validated in extended model systems.

## Materials and methods

### Osteosarcoma cell lines, xenografts, patient samples and normal controls

A panel of human osteosarcoma cell lines (*n* = 21) composed of 143B, CAL-72, G-292, HAL, HOS, IOR/MOS, IOR/OS9, IOR/OS10, IOR/OS14, IOR/OS15, IOR/OS18, IOR/SARG, KPD, MG-63, MHM, MNNG/HOS, OHS, OSA, Saos-2, U-2 OS and ZK-58 were obtained from ATCC (www.lgcstandards-atcc.org), DSMZ (http://www.dsmz.de/) or research partners in the EU funded EuroBoNeT project [50]. These cell lines have been extensively characterised at the molecular and phenotypic level [10, 51] and authenticated through STR testing.

The xenograft cohort comprised osteosarcoma xenografts established at the Norwegian Radium Hospital (*n* = 19) and at the University of Valencia (*n* = 22) [52, 53]. In short, human tumours were implanted subcutaneously in nude mice and passed successively. These xenografts have previously shown to be good representative models for osteosarcoma, showing gene expression profiles similar to their original tumour [54].

The osteosarcoma patient sample panel comprised fresh-frozen tissue from 14 high-grade osteosarcoma samples collected at the Norwegian Radium Hospital. The tumours were diagnosed by an osteosarcoma pathologist, according to the current World Health Organization classification [55].

Six bone samples were used as normal controls. Normal bones were purchased from Capital Biosciences (Capital Biosciences, MD, USA) (*n* = 2) or obtained from amputations of cancer patients at the Norwegian Radium Hospital (*n* = 4), where bone samples were collected distant from the tumour margin. DNA copy number analysis of the latter four samples (Bone 1–4) showed normal diploid karyotype [10].

### miRNA expression profiling

Total RNA from osteosarcoma cell lines and bones was extracted and quality controlled as previously described [24]. miRNA expression profiling was performed using the Agilent miRNA Complete Labeling and Hyb Kit Version 2.0, and Agilent Human miRNA Microarrays (version 2, 799 human miRNAs). miRNA data was imported into GeneSpring GX10 (Agilent Technologies Inc., CA, USA), and the intensity values were log_2_ transformed and quantile normalized. MIAME (minimum information about a microarray experiment) compliant data can be downloaded from the GEO repository (www.ncbi.nlm.nih.gov/geo/), accession number GSE28425.

### DNA methylation profiling

DNA was isolated using the Wizard Genomic DNA Purification Kit (Promega, WI, USA). For the initial analyses, DNA methylation profiling of 19 cell lines and four bone samples was performed using the Illumina HumanMethylation27 BeadChip (Illumina Inc., California, USA), covering 27,000 CpG sites across the genome [10]. For an extended validation, DNA methylation profiling was performed on 10 additional patient samples and four bone samples using the Infinium HumanMethylation450 BeadChip from Illumina, covering 485,000 CpG sites across the genome. Data extraction and initial quality control of the bead summary raw data were performed using GenomeStudio V2011.1 and the Methylation module v1.9.0, both provided by Illumina. For each sample, Beta values for each probe (average ratio of signal from methylated probe relative to the sum of both methylated and unmethylated probes) were exported for downstream analysis.

### Integration of miRNA and methylation data

The association between CpG methylation and miRNA expression data in osteosarcomas and normal samples was calculated using Pearson’s Correlation between miR-486-5p expression and methylation level (Beta) using the Methylation module v1.9.0 of GenomeStudio. DeltaBeta values for the groups of samples were calculated (Beta for cell lines minus Beta for normal samples).

### Quantitative real-time reverse-transcription PCR

qRT-PCR was performed using the ABI PRISM 7500 DNA Sequence Detection System (Life Technologies, CA, USA). TaqMan MicroRNA Reverse Transcription Kit and TaqMan MiRNA Assays (Life Technologies) were used to generate cDNA and to quantitatively detect the expression of mature miR-486-5p. For pri-miRNA hsa-mir-486-1 and *ANK1* expression quantification, the Fast Cells-to-Ct reagents (Life Technologies) were used to generate cDNA. Transcript-specific TaqMan Gene Expression Assays (Life Technologies) were used for quantitative PCR. Gene expression was normalized towards *RNU44* for miRNA quantification and glyceraldehyde 3-phosphate dehydrogenase (*GAPDH*) for pri-miRNA hsa-mir-486-1 and *ALK1* quantification. Overview of TaqMan assays are found in Additional file 1: Supplementary Table S1. The relative expression levels were determined using the comparative C_T_ method.

### 5-Aza-2′-deoxycytidine treatment

Osteosarcoma cells were cultured in the presence of 1 μM 5-Aza (Sigma-Aldrich MO, USA), as previously described [10]. The cells were harvested after 72 h and total RNA was isolated using the miRNeasy Mini Kit (Qiagen, Germany).

### DNA extraction and bisulfite conversion

Genomic DNA from tumour and normal samples was isolated by standard phenol chloroform extraction or using the Wizard Genomic DNA Purification Kit (Promega). DNA (1.3 μg) was bisulfite treated using the EpiTect Bisulfite Kit (Qiagen) and purified using the QIAcube (Qiagen).

### Qualitative methylation-specific polymerase chain reaction

Primers for MSP were designed using Methyl Primer Express 1.0 (Life Technologies) (Additional file 1: Supplementary Table S2). The MSP was carried out using approximately 24 ng of bisulfite treated DNA and performed as previously described [56]. All results were verified with a second round of MSP and scored independently by two of the authors.

### Direct DNA bisulfite sequencing

A subset of the osteosarcoma cell lines was subjected to direct bisulfite sequencing as previously described [57], allowing for a semi-quantitative visualization of 5-methylcytosines. The *mir-486* primers were designed using the Methyl Primer Express 1.0 (Life Technologies) and flanked the MSP amplicons (Additional file 1: Supplementary Table S2). The approximate degree of methylation at each CpG site was calculated by comparing the peak height of the cytosine signal to the sum of cytosine and thymine peak height signals as previously described [58]. CpG sites with ratios between 0 and 0.2 were classified as unmethylated, CpG sites with ratios in the range of 0.21–0.8 were classified as partially methylated, and CpG sites with ratios from 0.81–1.0 were classified as hypermethylated.

### Quantitative methylation-specific polymerase chain reaction

A quantitative qMSP analysis was carried out using approximately 30 ng of bisulfite-treated DNA and performed as previously described [56]. Commercially available fully methylated DNA (CpGenome Universal Methylated DNA, MA, USA), unconverted and bisulfite-treated normal DNA were included as controls. Primers and probes were designed using Primer Express Software 3.0 (Life Technologies) (Additional file 1: Supplementary Table S2). The *ALU-C4* repetitive element was used as an internal reference, and the values were calculated as percent of methylated reference, PMR [59]. The median gene: Alu ratio of each gene was divided by the median gene: Alu ratio of the positive control and multiplied by 100. To ensure high specificity for each qMSP assay, the thresholds for scoring the osteosarcoma samples as methylated were set according to the highest PMR value across the test series of normal bone. Samples with PMR values equal to or above the scoring threshold were considered to be methylated.

### Transfection with synthetic miRNAs and live cell imaging

Cells were seeded at a density of 1250–2500 cells per well in 96-well plates the day before transfection. Synthetic miR-486-5p mimics and Negative Control #1 (PM10546 and AM17110, respectively, Life Technologies) were transiently transfected into osteosarcoma cells at a final concentration of 15 nM using the INTERFERin siRNA transfection reagent (Polyplus-transfection SA, France). The cellular growth after transfection was measured every 3 h, by a live-cell imaging system, IncuCyte ZOOM (Essen Bioscience, UK) and the percent of cells confluence was calculated with the corresponding software application (version 2013BRev1).

### Statistics

Analyses were performed with the GraphPad Prism 9 software. Non-parametric Mann-Whitney U test was applied to compare groups of samples. *p*-values were derived from two-sided tests using a significance level of 0.05. Similarities between samples were calculated using Pearson’s correlation (r). Statistical differences between the group of cell lines before and after 5-Aza treatment were calculated using a paired t-test.

## Supplementary Information

**Additional file 1: Supplementary Table S1.** Overview of TaqMan assays for miRNA and mRNA qRT-PCR experiments. **Supplementary Table S2.** Primers used for qualitative Methylation-Specific Polymerase Chain Reaction (MSP), bisulfite sequencing (BS) and quantitative Methylation-Specific Polymerase Chain Reaction (qMSP). **Supplementary Figure S1.** Genomic overview of the *mir-486*/*ANK1* locus. **Supplementary Figure S2.** Expression of *ANK1* transcript variants in untreated and 5-Aza treated osteosarcoma cell lines. **Supplementary Figure S3.** Methylation level of miR-486 as assessed by methylation specific PCR (MSP).

## Data Availability

For the microarray experiments, MIAME compliant data can be downloaded from the GEO data repository (www.ncbi.nlm.nih.gov/geo/). The Agilent Human miRNA Microarray datasets are deposited under the accession number GSE28425, the Illumina HumanMethylation27 datasets under the accession number GSE36002 in SuperSeries number GSE36004 and the Infinium HumanMethylation450 DNA methylation datasets under the accession number GSE161407.
