# Supplementary material for: miR-486-5p expression is regulated by DNA methylation in osteosarcoma
Source: BMC Genomics. 2022 Feb 17;23:142. doi: 10.1186/s12864-022-08346-6 (PMC8851731; doi:10.1186/s12864-022-08346-6)
Supplement: Supplementary file 1 — Additional file 1: Supplementary Table S1. Overview of TaqMan assays for miRNA and mRNA qRT-PCR experiments. Supplementary Table S2. Primers used for qualitative Methylation-Specific Polymerase Chain Reaction (MSP), bisulfite sequencing (BS) and quantitative Methylation-Specific Polymerase Chain Reaction (qMSP). Supplementary Figure S1. Genomic overview of the mir-486/ANK1 locus. Supplementary Figure S2. Expression of ANK1 transcript variants in untreated and 5-Aza treated osteosarcoma cell lines. Supplementary Figure S3. Methylation level of miR-486 as assessed by methylation specific PCR (MSP). [file 12864_2022_8346_MOESM1_ESM.docx]

**Supplementary Table S1**

| **Overview of TaqMan assays for miRNA qRT-PCR experiments** | | |  |
| --- | --- | --- | --- |
|  |  | |  |
| **miRNA** | **AssayID** | **Mature sequence** |  |
| miR-486-5p | 1278 | UCCUGUACUGAGCUGCCCCGAG |  |
|  |  |  |  |
| **pri-miRNA** | **AssayID** | Stem-loop sequence |  |
| hsa-miR-486-1 | Hs03303815_pri | GCAUCCUGUACUGAGCUGCCCCGAGGCCCUUCAUGCUGCCCAGCUCGGGGCAGCUCAGUACAGGAUAC |  |
|  |  | |  |
| **Endogenous control** |  | |  |
| **Assay** | **AssayID** | **Control sequence** |  |
| *RNU44* | 1094 | CCTGGATGATGATAGCAAATGCTGACTGAACATGAAGGTCTTAATTAGCTCTAACTGACT |  |
|  | | |  |
| **Overview of TaqMan assays for mRNA qRT-PCR experiments** | | |  |
| **mRNA** | **AssayID** | **Transcript variant** |  |
| *ANK1* | Hs00986630_m1 | Variant 1-4 |  |
| *ANK1* | Hs00986660_m1 | Variant 9 |  |
| *ANK1* | Hs00252830_m1 | Variant 5,7,10 (sANK1). |  |
| *GAPDH* | Hs99999905_m1 |  |  |

**Supplementary Table S2**

**Primers used for qualitative Methylation-Specific Polymerase Chain Reaction (MSP) and bisulfite sequencing (BS).**

| **Primer Set** | **Forward Primer** | **Reverse Primer** | **Fragment size** | **Fragment Location** | |
| --- | --- | --- | --- | --- | --- |
| *mir-486* MSP-M | GTAATTTGAGGTTTTTAGGC | ACTAACGAACTCACCGCA | 152 | -128 to +24 |  |
| *mir-486* MSP-U | TTTGTAATTTGTGGTTTTTAGGT | AACTAACAAACTCACCACAAC | 156 | -131 to +25 | |
| *mir-486* BS | GTGGTTTGATGTTTAATTTTGG | TAACCCCCTCCTAACATCTC | 424 | -261 to +163 | |

Abbreviations: MSP, methylation-specific polymerase chain reaction; BS, bisulfite sequencing; M, methylated-specific primers; U, unmethylated-specific primers; Frg. Size, fragment size (in bases); Fragment location indicates the start and end point (in bases, provided by the USCS Genome Browser) of each fragment relative to the transcription start site of *ANK1* variant 1-4 for *mir-486*.

**Primers and probe used for quantitative Methylation-Specific Polymerase Chain Reaction (qMSP).**

| **Assay** | **Forward Primer** | **Reverse Primer** | **Probe** | **Fragment size** |
| --- | --- | --- | --- | --- |
| *mir-486* | TTAGGCGGGCGTTATTTTTTC | GTACGAACCAAACCCCCGA | TTCGGTTCGATAGTAAGC | 100 |


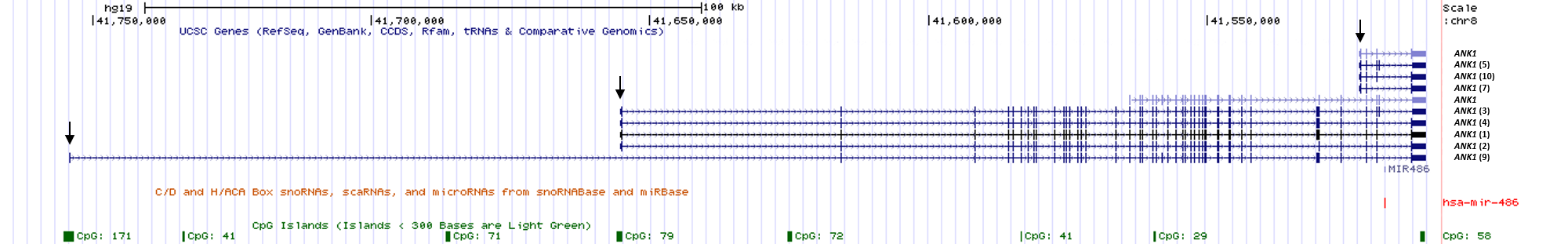


**Supplementary Figure S1. Genomic overview of the** *mir-486*/*ANK1* locus. Shown is different *ANK1* isoforms with TSS (vertical arrow), hsa-miR-486 and the CpG islands in the region. Modified from UCSC Genome Browser NCBI, GRCh37/hg19 assembly.

**
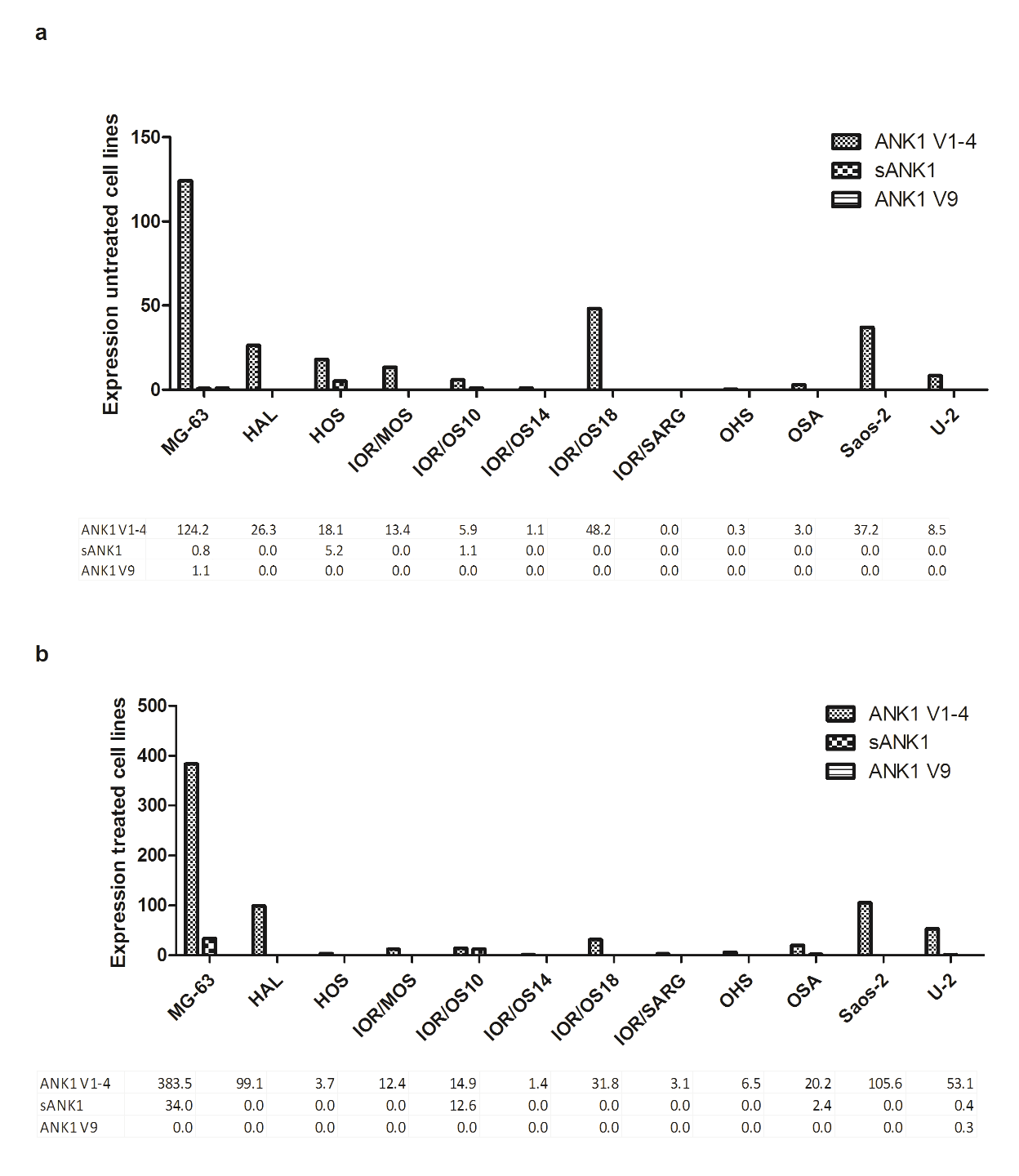
**

**Supplementary Figure S2. Expression of *ANK1* transcript variants in untreated and 5-Aza treated osteosarcoma cell lines.** Quantification of *ANK1* using qRT-PCR in cell lines. **a.** Expression of untreated cells. **b.** Expression of 5-Aza treated cells. Expression normalized against GAPDH. Cell lines with undetected expression is set to 0.

|  | **CGI CpG79**  **variant 1-4** | **CGI CpG171**  **variant 9** |
| --- | --- | --- |
| 143B | **M** | **U/M** |
| Cal72 | **M** | **U** |
| G-292 | **U/M** | **U** |
| GCT | **U/M** | **U** |
| HAL | **M** | **U** |
| HOS | **M** | **U** |
| IOR/OS9 | **U/M** | **U** |
| IOR/OS10 | **M** | **U** |
| IOR/OS14 | **U** | **U** |
| IOR/OS15 | **U/M** | **U** |
| IOR/OS18 | **M** | **U** |
| IOR/MOS | **M** | **U** |
| IOR/SARG | **M** | **U** |
| KPD | **M** | **U** |
| MG-63 | **M** | **U** |
| MHM | **U/M** | **U** |
| MNNG/HOS | **U/M** | **U** |
| OHS | **U/M** | **U** |
| OSA | **M** | **U** |
| Saos-2 | **U/M** | **U** |
| U2- OS | **M** | **U** |
| ZK58 | **U/M** | **U** |
|  | **21/22** | **1/22** |

**Supplementary Figure S3. Methylation level of miR-486 as assessed by methylation specific PCR (MSP).** MSP analyses of the CGI upstream of *miR-486,* covering the TSS of *ANK1* variant 1-4 and *ANK1* variant 9. MSP was performed on 21 osteosarcoma cell lines. M, methylated; U/M,: presence of both methylated and unmethylated band; U, unmethylated.
